# Systematic Review Regarding the Clinical Implications of Allograft and Alloplastic Bone Substituents Used for Periodontal Regenerative Therapy

**DOI:** 10.3390/jcm14030894

**Published:** 2025-01-29

**Authors:** Alexandru Vlasa, Eugen Bud, Luminita Lazăr, Souiah Ilies, Alexandra Mihaela Stoica, Ana-Petra Lazăr, Ioana Martu, Anamaria Bud

**Affiliations:** 1 Department of Periodontology and Oral-Dental Diagnosis, Faculty of Dental Medicine, George Emil Palade University of Medicine and Pharmacy, Science, and Technology, 540139 Târgu-Mureș, Romania; alexandru.vlasa@umfst.ro (A.V.); luminita.lazar@umfst.ro (L.L.); 2 Department of Orthodontics and Dental-Facial Orthopedics, Faculty of Dental Medicine, George Emil Palade University of Medicine and Pharmacy, Science, and Technology, 540139 Târgu-Mureș, Romania; 3 Independent Researcher, 75012 Paris, France; souiah.ilies@icloud.com; 4 Department of Odontology and Oral Pathology, Faculty of Dental Medicine, George Emil Palade University of Medicine and Pharmacy, Science, and Technology, 540139 Târgu-Mureș, Romania; alexandra.stoica@umfst.ro; 5 Department of Oral Rehabilitation and Occlusology, Faculty of Dental Medicine, George Emil Palade University of Medicine, Pharmacy, Science, and Technology, 540139 Târgu-Mureș, Romania; ana.lazar@umfst.ro; 6 Department of Oral Implantology, Removable Dentures and Technology, Faculty of Dental Medicine, “Grigore T. Popa” University of Medicine and Pharmacy, 700115 Iasi, Romania; 7 Department of Pedodontics, Faculty of Dental Medicine, George Emil Palade University of Medicine and Pharmacy, Science, and Technology, 540139 Târgu-Mureș, Romania; anamaria.bud@umfst.ro

**Keywords:** periodontal regeneration, alloplastic bone, allograft bone, systematic review

## Abstract

**Background/Objectives:** Regenerative periodontal therapy is a treatment method that focuses on restoring the periodontium affected by chronic inflammatory disease or injury. It involves using different biomaterials and techniques to completely restore the periodontal structures. The main objective was to identify and critically evaluate relevant studies comparing the clinical efficacy of allograft and alloplastic materials in regenerative periodontal therapy. **Methods:** For evaluation, a systematic review based on PRISMA guidelines was conducted. Data were extracted using only specific types of study designs, which included randomized controlled trials, cohort studies, and case-control studies. Target patients with periodontal disease or periodontal lesions undergoing periodontal therapy using allograft or alloplastic materials were selected. Periodontal parameters such as clinical attachment level, probing pocket depth, radiographic bone fill, or patient-reported outcomes were analyzed. **Results:** The results showed that allograft and alloplastic materials offered reduced pocket depth, a gain in clinical attachment, and bone repairment. The variation observed indicated that allografts showed a slightly more significant clinical attachment gain and a superior bone fill than alloplastic ones, suggesting that allografts enhance osteogenesis and provide a greater capacity for repair in periodontal defects. **Conclusions:** The results of the present study suggest that allograft and alloplastic materials offered reduced pocket depth, a gain in clinical attachment, and bone repairment, with both methods having similar clinical efficacy.

## 1. Introduction

Regenerative periodontal therapy is a treatment method that focuses on restoring the periodontium affected by chronic inflammatory disease or injury. It involves using different biomaterials and techniques to completely restore the attachment apparatus (the structure and function) [1]. This approach uses surgical procedures, like guided tissue regeneration and flap surgery, and biomaterials, such as bone grafts, growth factors, and membranes [2]. According to the new guidelines, regenerative surgery is part of step 3 [3,4,5]. Moreover, it induces complete regeneration of the periodontal tissue and is applied not only in cases of chronic damage, but also in acute conditions. This treatment is recommended for patients with advanced periodontitis, or other periodontal defects that cannot be effectively treated with non-surgical methods (for individuals who are suffering from progressive periodontitis or other periodontal deficiencies that cannot be remedied through non-invasive means) [6]. Allografts refer to tissues obtained from individuals of the same species (other humans, in this case) [6,7,8]. They are often used in bone regeneration procedures and are available in large amounts. Allografts do not possess the typical limitations associated with autografts, which are tissues harvested from the same individual. Allografts come in various forms, such as cancellous and cortical of different sizes [8]

Allografts, used for periodontal procedures, come in the form of cortical wedges, cortical chips, cortical granules, and powdered cancellous bone [6]. These are prepared in various ways, including frozen, freeze-dried, mineralized, or demineralized. These materials not only function as osteoconductive substances, but they also might possess some osteo-inductive capability, given the presence of bone morphogenetic proteins (BMP) [9]. 

Bone morphogenetic proteins (BMPs) are vital in promoting bone growth. These BMPs are essential factors that facilitate the process of bone formation. BMP is a growth factor for undifferentiated cells, including bone-forming cells [10] They increase the activity of the alkaline phosphatase enzyme (a specific enzyme for bone cells) [3,11,12]. They also attract cells like monocytes to attach to a part of the extracellular matrix called type IV collagen. However, the main issue is the risk of disease transmission associated with allografts. Despite strict screening procedures and the use of virucidal methods during tissue processing, a small risk remains [6]. The possibility of tissue contamination, and the transmission of diseases from newly identified pathogens, presents inherent risks, as existing donor screening and tissue processing strategies may not eliminate these risks. It is essential to stay vigilant and continually update screening procedures to minimize the potential for disease transmission [9,10,13].

On the other hand, alloplastic refers to synthetic bone graft products used in dental procedures, including periodontal and bone regeneration [14]. Alloplastic bone refers to synthetic materials that contain some of the essential chemical components of natural bone (e.g., calcium and phosphate) and are known to promote bone regeneration. Alloplastic bone substitutes are often preferred because they are readily available, have a high safety standard, and can be easily molded [15]. They also have an adequate resorption rate, which means they can be biodegraded and replaced by new bone over time. Alloplastic materials have osteoconductive capabilities, which means they can support the growth of new bone [16]. Alloplastic fillers such as Hydroxy-apatite, polymers, and microspheres are widely used in the practice: experiments have been conducted with various therapeutic approaches, and current scientific interest in alloplastic replacements is focused primarily on calcium phosphate ceramics [7,8].

The null hypothesis of the present research was that there is no difference in regard to the clinical efficacy between allograft and alloplastic materials used for reconstructive periodontal therapy. Amid its gaining popularity in clinical periodontics, our current understanding of treatment effectiveness, efficiency, and stability needs to be reassessed. The literature shows a lack of knowledge and data regarding a clinical comparison between these regenerative materials in the periodontology community, yet many clinicians continue to utilize the devices in practical or educational settings.

This review would offer a comprehensive and evidence-based assessment of the relative clinical efficacy of allograft and alloplastic materials used in regenerative periodontal therapy, aiming to identify and critically evaluate relevant studies published in the literature. The clinical outcomes, such as changes in probing depth, clinical attachment level, and radiographic bone fill, reported in studies using allograft and alloplastic materials, were also evaluated.

## 2. Materials and Methods

The present study was carried out following the “Preferred Reporting Items for Systematic Reviews and Meta-Analyses” or PRISMA guidelines [17]. Prospective registration of this systematic review was submitted to PROSPERO™ for registration (registration number CRD42023470779 19 October 2023). Authors chose PICO framework in order to achieve the objectives of the study. It is the most commonly used model for structuring clinical analyses and questions.

P (patient, problem)—adult human patients with no previous orthodontic therapy in the medical history, no general disorders associated

I (intervention, exposure)—periodontal regenerative therapy

C (comparison)—alloplastic versus allograft regenerative materials

O (outcomes)—clinical attachment level, probing pocket depth, radiographic bone fill, or patient-reported outcomes at the regular post-surgery check-ups

An electronic bibliographic search was carried out between 1 July 2024 and 31 August 2024 in the databases Medline (via PubMed), Scopus, and Cochrane. For evaluation and extraction of data, only specific types of study designs were selected, such as randomized controlled trials, cohort studies, and case-control studies. The target was set to select only patients with periodontal disease or periodontal lesions undergoing periodontal therapy using allograft or alloplastic materials. The authors considered all publications that had been published in the literature in relation to the topic of the present study, and the number of papers found was quite limited. Clinical attachment level, probing pocket depth, radiographic bone fill, or patient-reported outcomes were also evaluated.

### 2.1. Inclusion Criteria

Articles that featured material relevant to the review’s objectives and that covered all age groups were chosen for full-text screening. The authors considered including articles that presented randomized/non-randomized investigations, clinical cases with large sample sizes, in-depth case reports, and validated comparative analyses. The year of the publication was not a criterion in the selection process.

### 2.2. Exclusion Criteria

Only articles written in English were selected. Studies involving animal subjects, seminar or conference presentations, academic publications, opinion pieces, and incomplete data were not included in the scope of the examination.

The authors did not limit their search based on the research publication dates. Instead, they took into account all publications that had been released in relation to our topic, as the number of papers found that matched the review criteria was limited.

### 2.3. Data Selection Protocol

Two separate reviewers combed through relevant publications in databases using specific keywords as Medical Subject Heading (Mesh), such as “allograft”, “Periodontal tissues regeneration”, and “alloplastic”. The same two reviewers separately extracted the following information after selecting the articles: authors, year of publication, type of publication, study topic, population demographics (n, age), outcome measure(s), pertinent result(s), and conclusion(s). To evaluate the methodological quality of included studies, the articles’ data were independently evaluated by the authors using a special manual form designed according to the following categories: study model design, number of subjects, and study results. The selected articles were compared. In case of a disagreement, a third reviewer was consulted. A second screening of the full text of the remaining studies was then performed to confirm their eligibility. Subsequently, a screening of the references of the included studies was performed to look for additional potentially eligible studies.

### 2.4. Quality and Risk-of-Bias Assessment

**Figure 1:**
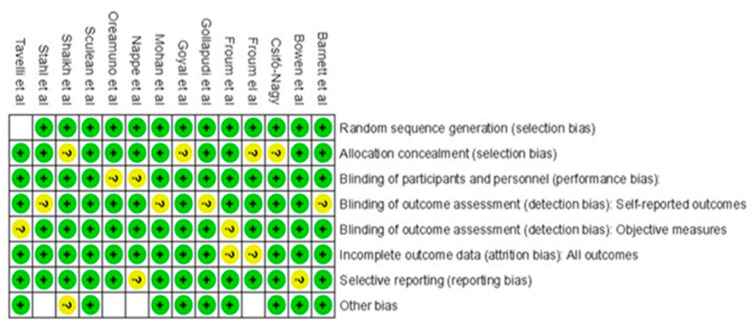
Revman Cochrane risk-of-bias assessment conducted during the study [18,19,20,21,22,23,24,25,26,27,28,29,30,31].

The quality assessment of the included studies was performed using the Revman Cochrane™, (The Cochrane Collaboration, London, United Kingdom) approach (Figure 1). The risk-of-bias tool identifies the domains specified in the Cochrane risk-of-bias instruments for systematic reviews. All the authors [18,19,20,21,22,23,24,25,26,27,28,29,30,31] clearly defined both their study objective and the population (number, characteristics, and eligibility) on which they were going to carry out the research.

### 2.5. Data Selection

**Figure 2:**
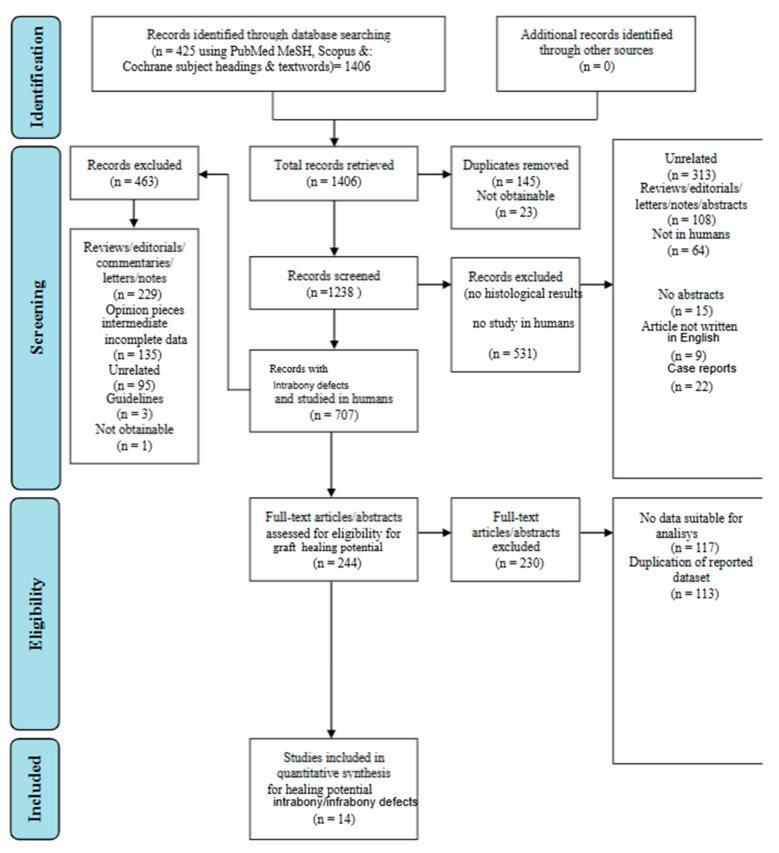
Flowchart representation of selection of articles through research framework [32].

A thorough search of the online journals turned up 1406 documents in total. The references yielded from the search strategy were exported to Microsoft Excel™ (version 2019 for Windows). Duplicates were manually discarded using this software. After discarding duplicates, a first screening was performed based on article titles and abstracts of the articles, following the inclusion/exclusion criteria described previously. After the removal of 145 articles that were identical or duplicates, 463 not fulfilling the review inclusion criteria, and 896 articles considered irrelevant for the purpose of this paper, only 244 original papers remained. A further set of 230 articles were excluded after the abstracts and titles of the submissions were examined. Ultimately, 14 documents—mostly clinical cases, in-vivo experiments, and comparative analyses in humans—were chosen that satisfied both the essential inclusion and exclusion criteria (Figure 2).

## 3. Results

**Figure 3:**
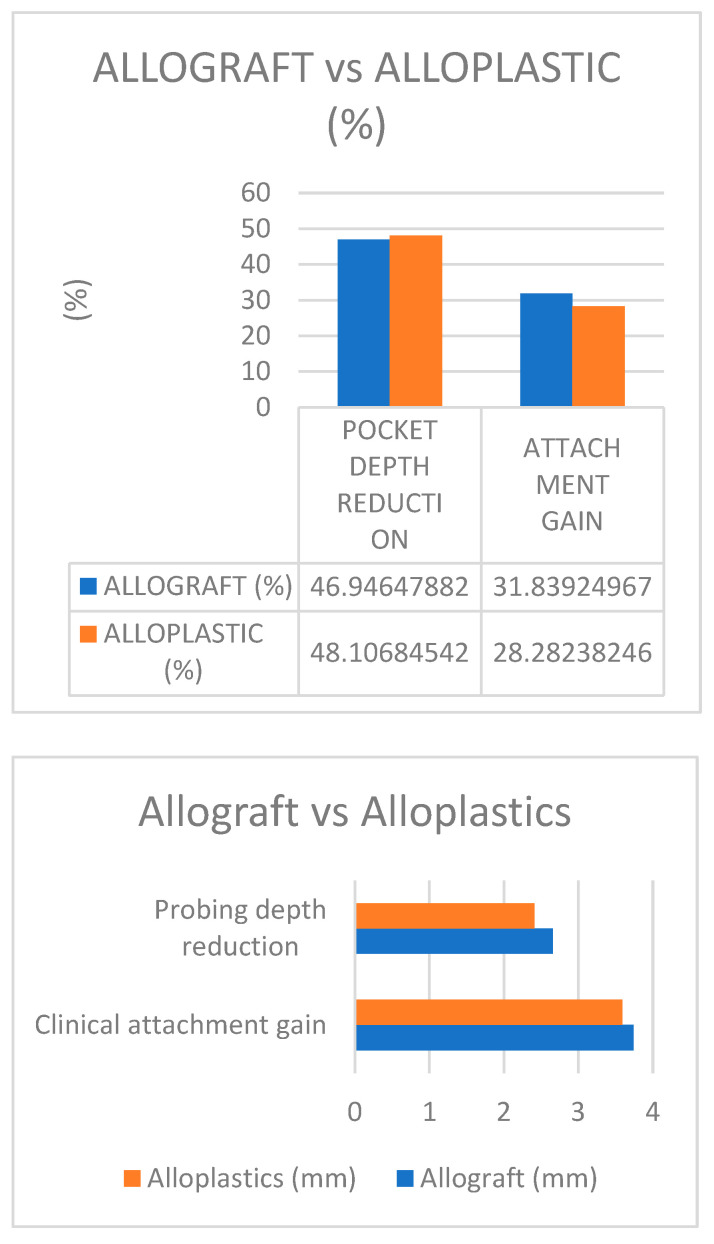
Comparative assessment of the studies included within research framework; Blue color-Allograft; Orange color-Alloplastic.

Results shown in Table 1, Figure 3 provide a comprehensive overview of the findings of the present study. On average, a negligible difference of 1% in probing depth reduction, and 3% in clinical attachment gain between allograft and alloplastic studies, were observed [22,27,28,30,31].

Similar results were found by previous researchers when clinically comparing the healing potential of the osteo-inductive decalcified freeze-dried bone allograft (DFDBA) with the osteo-conductive alloplastic synthetic graft, particulate porous Hydroxyapatite (HA), bone morphogenetic proteins (BMPs), and biphasic calcium phosphate (BCP) at 12 months (Table 2) [19,23]. 

**Figure 4:**
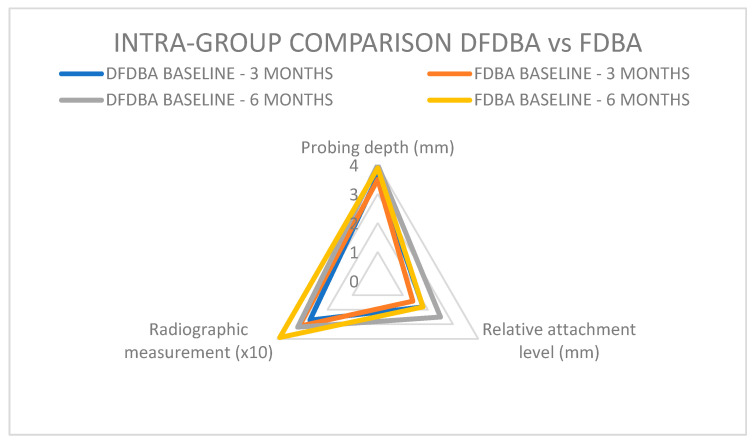
Intra-group comparison DFDBA vs. FDBA.

**Figure 5:**
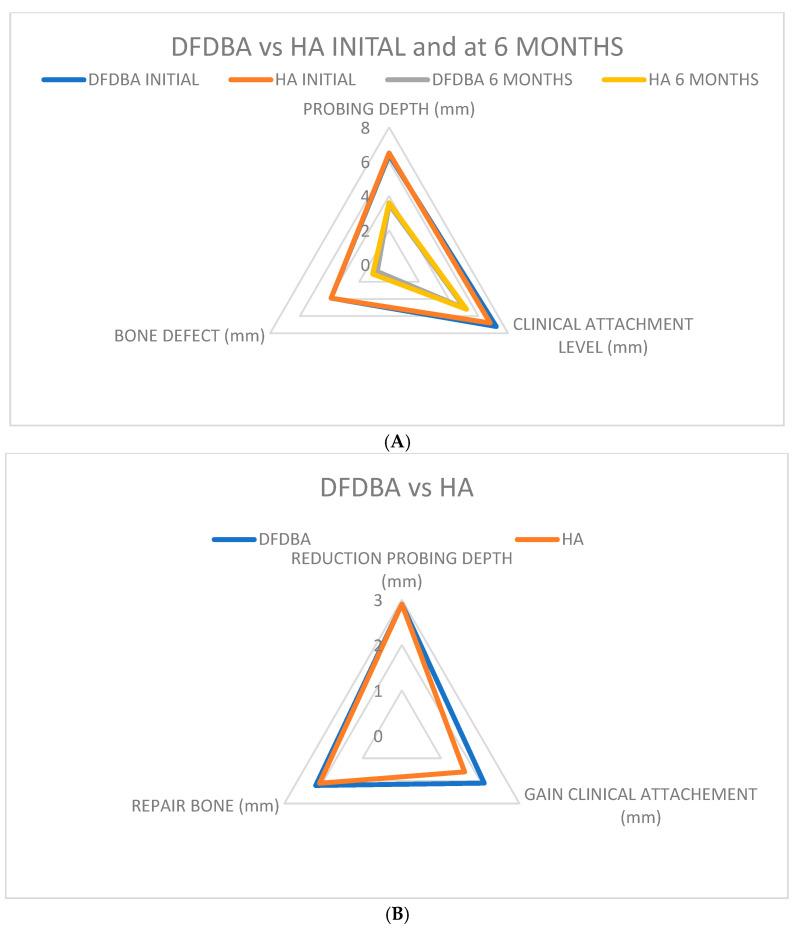
(**A**) Comparative results at six months regarding clinical attachment level. (**B**) Comparative results at six months regarding probing depth reduction.

When comparing decalcified freeze-dried bone allograft (DFDBA) with porous Hydroxyapatite (HA) alloplastic, previous studies [22,27,30] showed that the average recession was 0.6 mm more in sites treated with Hydroxyapatite than those treated with decalcified freeze-dried bone allograft (DFDBA). Both grafts resulted in a reduction in probing depth by 2.9 mm (Figure 4 and Figure 5A,B) at six months post-treatment.

**Figure 6:**
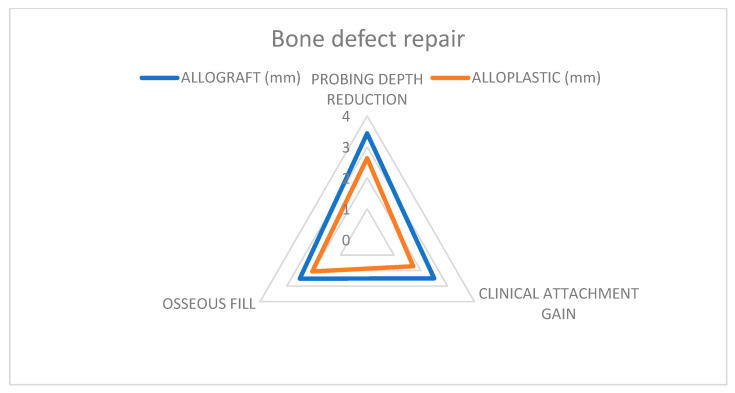
Comparative clinical attachment gain results; Blue color—allograft; Orange color—alloplastic.

The gain in clinical attachment level was slightly higher in DFDBA-treated sites (2.1 mm) compared to Hydroxyapatite-treated sites (1.6 mm) (Figure 6) at 6 months. However, these differences in soft tissue measurements were not statistically significant (*p* < 0.05). Similarly, the paired *t*-test did not reveal significant differences between subjects (*p* < 0.05).

As for bone defect repair, DFDBA-treated sites showed a slightly higher repair (2.4 mm) than Hydroxyapatite-treated sites (1.9 mm), but again, these differences were not statistically significant. (*p* < 0.05). Both materials offered a reduced pocket depth and a gain in clinical attachment (Figure 6).

**Figure 7:**
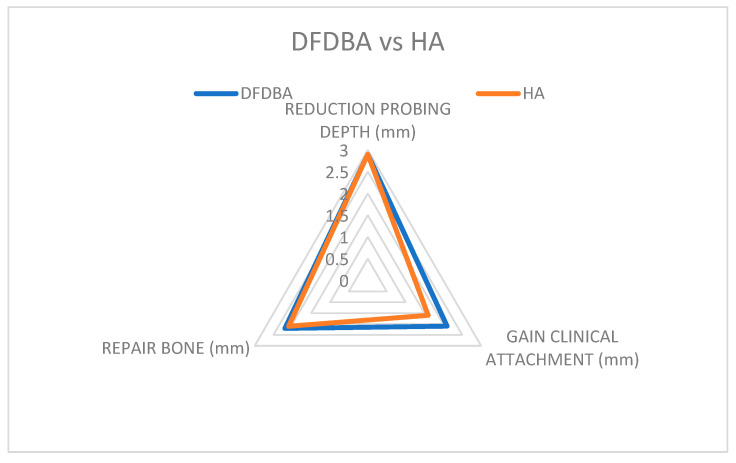
Comparative clinical attachment gain results; Blue color—DFDBA; Orange color—HA.

There were no statistically significant differences observed when comparing bone repair results. The studies [22,23,27] concluded that DFDBA and Hydroxyapatite effectively treat osseous defects, with no significant difference in effectiveness (Figure 7).

**Figure 8:**
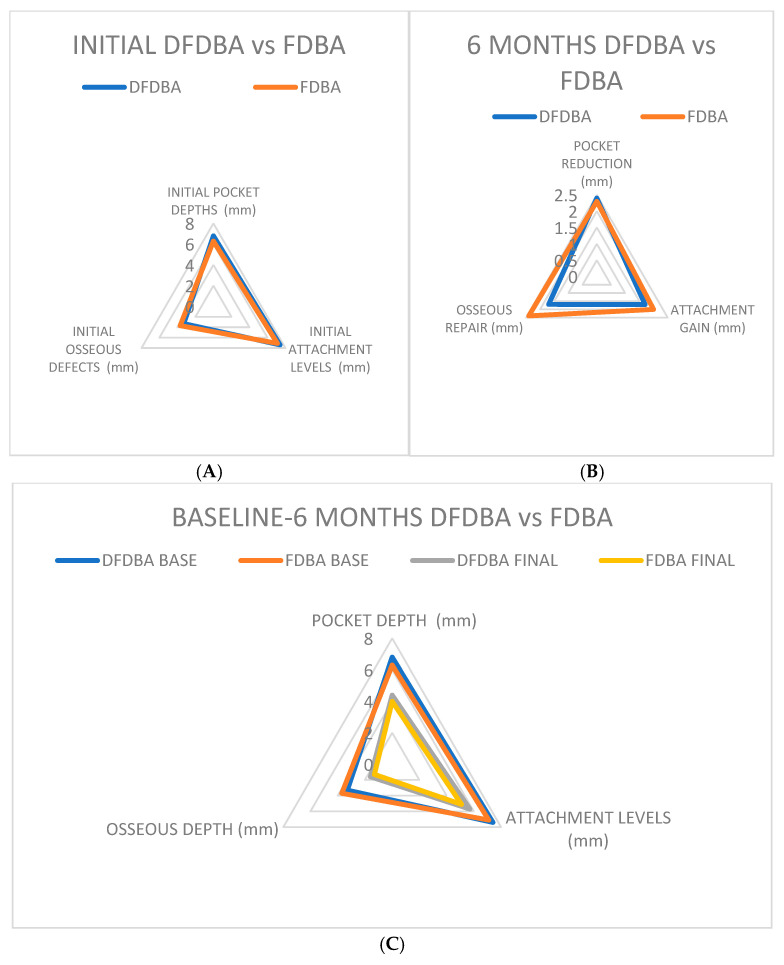
Intragroup comparison of the studies; (**A**) initial pocket depth DFDBA/FDBA; (**B**) 6 months pocket depth reduction DFDBA vs. FDBA; (**C**) baseline–6 months after treatment DFDBA vs. FDBA pocket reduction.

When comparing data regarding DFDBA and FDBA [33,34], the following results (Figure 8A–C) were obtained.

When assessing the impact of biphasic calcium phosphate (BCP) and demineralized freeze-dried bone allograft (DFDBA) (Table 3) on pocket probing depth, with evaluations performed both clinically and radiographically, previous studies [20,23] showed significant results at three months (*p* value < 0.001). No significant results were observed at six months (*p* > 0.001).

**Figure 9:**
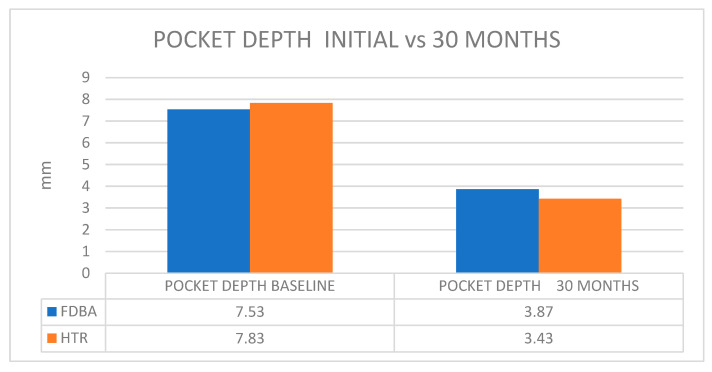
Mean values of pocket depth reduction at baseline vs. 30 months; Blue color—FDBA; Orange color—HTR.

Further studies [27,28] conducted over the long term (30 months), regarding the outcomes of utilizing Hard Tissue Replacement Synthetic Bone (HTR) and freeze-dried bone allograft (FDBA), concluded that the results were similar regarding the clinical attachment level changes after 30 months post-treatment (Table 4, Figure 9).

## 4. Discussion

Studying periodontal regeneration through the clinical examination of human samples comes with technical difficulties, including the challenges of interpreting the data. However, this approach is crucial as it offers valuable insights into the biological potential of different regenerative methods and materials. This knowledge is critical to progressing the research and clinical practice in this field. Therefore, the findings from these studies should be interpreted cautiously, considering the evidence from pre-clinical and clinical studies designed to validate the concept.

Database searches show a lack of literature regarding this subject, and most of the studies lack power due to multiple biases. None of those studies prove the superiority of one method over the other one. Most authors mentioned that it acts as a basic “filler” without offering osteogenesis or promoting it [20,23,25]. Other studies mentioned that debridement alone offers the same results as alloplastic [19]. These findings may be explained by the influence of various factors, including the morphological traits and dimensions of naturally developing periodontal defects, which can impact the results of these studies [21]. 

Applying biomaterials, including allografts and alloplastic materials, has demonstrated encouraging outcomes in advancing periodontal regeneration. However, the dental community has yet to agree on the best choices and application methods for these biomaterials in a clinical setting. By expanding our understanding of regenerative periodontal therapy, we can potentially guide future research in this area. This could ultimately lead to improved treatment results for patients suffering from periodontitis and elevate the standard of care provided by dental professionals.

On the other hand, most of the authors did not mention the postoperative complications (one of the most important clinical parameters for the patient). The results of having a better attachment should not interfere with the patient’s good health and lifestyle. Many studies focus on the clinical outcomes and do not fully capture the patient-reported effect or other essential aspects of treatment efficacy to patients and clinicians. It is important to mention that clinical studies about allografts have widely stopped due to new materials, such as xenografts, and also due to some ethical reasons. However, the allograft remains one of the gold standards for grafts with autogenous bone.

Growth factors and bioactive proteins, like BMPs, transforming growth factor beta (TGF-beta), and insulin-like growth factors I and II (IGF-I and IGF-II), play a pivotal role in tissue regeneration by modulating cellular processes such as cell migration, proliferation, differentiation, and matrix synthesis. In periodontal therapy, harnessing these biological molecules can augment the natural regenerative capacity of the periodontium and improve treatment outcomes [4,13,18]. Recombinant human platelet-derived growth factor-BB (rhPDGF-BB) is a molecule that has gained significant attention for its role in periodontal regeneration. PDGF, a potent mitogen in the body, is secreted by platelets, macrophages, and endothelial cells, among others, and is crucial in promoting cell proliferation, chemotaxis, and angiogenesis. The BB homodimer, rhPDGF-BB, is the most potent PDGF isoform and has been used successfully in periodontal and peri-implant bone regeneration. It acts on cells involved in periodontal healing, including periodontal ligament cells, gingival fibroblasts, osteoblasts, and cementoblasts, stimulating their proliferation and migration [5].

The use of platelet concentrates, like Platelet-Rich Plasma (PRP) and Platelet-Rich Fibrin (PRF) [24,26,29] is another technique that leverages the natural growth factors found within platelets. These autologous sources are derived from the patient’s blood and can release many growth factors, including PDGF, involved in wound healing and tissue regeneration. Their application in periodontal therapy has shown promising results, particularly in procedures like periodontal pocket reduction and bone grafting. A very recent systematic review revealed that the utilization of the APC/PRF membranes, although a valid alternative to the traditional CTG, showed inferior results in terms of RC. Prospective investigations, including analysis of GT and KTW, are required since they have been demonstrated as significant prognostic factors for long-term outcomes [13]. In a very similar way, clinical in vivo studies demonstrated that even extensive periodontal defects transplanted with a bone substitute plus L-PRF and guided tissue regeneration exhibited regeneration, given enough time for tissue remodeling and maturation [3,7,8]. Guided tissue generation technique (GTR), barrier membranes, or grafting materials and L-PRF combined with these different techniques and biomaterials, acted in synergy and had a significant positive impact on bone regeneration, especially in horizontal bone loss [39]. Therefore, the diminished bone growth seen in a number of biopsies may simply be the product of a short healing period [8]. 

The peptide P-15 [25] also known as an organic bovine-derived hydroxyapatite matrix (ABM)/P-15, is a synthetic peptide that mimics the cell-binding domain of collagen. It serves as a matrix that facilitates cell attachment and proliferation. When P-15 is combined with a matrix like ABM, it creates a three-dimensional structure that allows for cell colonization and rapid bone formation. P-15 has been shown to improve clinical outcomes in periodontal regenerative procedures [20]. In conclusion, bioactive molecules like rhPDGF-BB, platelet concentrates, and peptide P-15 represent promising tools for enhancing periodontal regeneration. They can stimulate the body’s natural healing processes, contributing to improved regeneration of periodontal tissues and better clinical outcomes. Nevertheless, further exploring these therapies with well-designed clinical trials is crucial to understand their potential and limitations in periodontal therapy [25,39,40,41,42].

More research is needed to explore long-term effectiveness. Improving clinical parameters via healing by the long junctional epithelium seems acceptable, but the goal is periodontal regeneration. Much research goes toward this objective, including research on bioactive agents such as recombinant human platelet-derived growth factors, platelet-rich plasma, enamel matrix derivative, and the P-15 peptide [43,44,45,46,47]. To obtain significant results for comparing the clinical parameters between alloplastic and allograft, future studies can conduct multicentric, prospective and randomized clinical trials. The study groups will have to be randomized in a block design, with stratification allowing each group’s coherence and comparability. Suggesting that allografts enhance osteogenesis and provide a greater capacity for repair in periodontal defects, authors can study if alloplastic materials act as a basic bone ‘filler’. The authors of the present study believe that the amount of regeneration observed may not necessarily reflect the strong biological potential of a technique or material, but could be influenced by the method of biopsy harvesting or other examinations, including histological evaluation.

## 5. Conclusions

The results of the present study suggest that allograft and alloplastic materials offered reduced pocket depth, a gain in clinical attachment, and bone repairment, with both methods having similar clinical efficacy. The difference in repair potential between allograft and alloplastic is minor or negligible. However, alloplastic is synthetic and naturally derived, offering availability, cost-effectiveness, and usage safety advantages.

## Figures and Tables

**Table 1:** Summary results of the studies analyzed using our methodology.

Study No	Authors	Material	Number of Patients	Number of Defects
1	Rummelhart et al. [33]	DFDBA vs. FDBA	9	22
2	Gothi et al. [34]	DFDBA vs. FDBA	10	20
3	Chandrashekar et al. [35]	Biograft-HT® Hydroxyapatite and β-tricalcium phosphate (70:30 ratio)	20	30
4	Kaushal et al. [36]	OSSIFI® Beta-tricalcium phosphate and Hydroxyapatite	10	10
5	Stavropoulos et al. [37]	Granular β-tricalcium phosphate	5	5
6	Horvath et al. [38]	Nanocrystalline hydroxyapatite (nano-HA)	6	6
7	S. S. Stahl and S. J. Froum [19]	Hydroxyapatite	3	12
8	S. S. Stahl and S. J. Froum [19]	Tricalcium phosphate	1	5
9	Bowen et al. [30]	Decalcified freeze-dried bone allograft (DFDBA) vs. Porous Hydroxyapatite (HA) alloplastic	6	34
10	Barnett et al. [31]	Freeze-dried bone allograft (FDBA) vs. Porous hydroxylapatite (HA)	7	38
11	Oreamuno et al. [22]	Decalcified freeze-dried bone (DFDBA) vs. porous hydroxyapatite (HA)	12	24
12	Kumar et al. [11]	Demineralized freeze-dried bone allograft (DFDBA) vs. Biphasic calcium phosphate (BCP)	42	42
13	S J Froum [27]	Freeze-dried bone allograft (FDBA) vs. HTR (Hard Tissue Replacement Synthetic Bone)	1	6
14	Stahl et al. [28]	ALLOGRAFT (NA) vs. ALLOPLASTIC (DURAPATITE)	1	2

**Table 2:** Comparative analysis regarding healing potential within study groups.

	Clinical Attachment Level (mm)			Probing Depth (mm)			Depth of Bone Defect (mm)		
	Initial	6–11 Months	Gain	Initial	6–11 Months	Reduction	Initial	6–11 Months	Fill
Allograft	6.8 ± 2.2	4.6 ± 1.4	2.2 ± 1.7	6.7 ± 2.1	3.7 ± 1.6	3.0 ± 2.0	8.1 ± 2.4	6.0 ± 1.6	2.1 ± 1.5
Alloplastic	6.9 ± 1.2	5.6 ± 1.4	1.3 ± 1.2	6.1 ± 1.5	4.6 ± 1.6	1.4 ± 1.7	7.6 ± 1.2	6.3 ± 2.0	1.3 ± 1.9

**Table 3:** Intragroup comparison of pocket probing depth values (in mm) at baseline, three months, and six months post-treatment.

Site	Time	n	Mean ± Sem	Mean Difference ± Sem	*p*-Value
BCP	Baseline	21	8.4 ± 0.29		
	Three months	21	5.9 ± 0.19	Baseline–3 Months: 2.5 ± 0.21	<0.001 *
	Six months	21	4.8 ± 0.17	Baseline–6 Months: 3.6 ± 0.23	=0.08
DFBA	Baseline	21	8.8 ± 0.29		
	Three months	21	5.2 ± 0.17	Baseline–3 Months: 3.6 ± 0.34	<0.001 *
	Six months	21	3.7 ± 0.10	Baseline–6 Months: 5.1 ± 0.28	=0.012

* Means statistically significant.

**Table 4:** Comparison of clinical data of FDBA and HTR grafted sites (mm).

	Probing Depth		Osseous Depth	Recession	Clinical Attachment Level Changes
	Baseline	30 Months	Baseline	30 Months	30 Months
FDBA					
	6.9	3.3	2.9	1.1	2.5
	7.5	3.8	3.2	1.5	2.2
	8.2	4.5	4.0	1.8	1.9
Average Mean ± Sem (Calculated)	7.53 ± 0.14	3.87 ± 0.13	3.37 ± 0.12	1.47 ± 0.08	2.20 ± 0.07
HTR					

## Data Availability

The original contributions presented in the study are included in the article, further inquiries can be directed to the corresponding author.
